# Proteomic analysis of a disease-resistance-enhanced lesion mimic mutant *spotted leaf 5* in rice

**DOI:** 10.1186/1939-8433-6-1

**Published:** 2013-01-07

**Authors:** Xifeng Chen, Shufang Fu, Pinghua Zhang, Zhimin Gu, Jianzhong Liu, Qian Qian, Bojun Ma

**Affiliations:** 1 College of Chemistry & Life Sciences, Zhejiang Normal University, Jinhua, 321004 China; 2 China National Rice Research Institute, Chinese Academy of Agricultural Sciences, Hangzhou, 310006 China

**Keywords:** Rice, Lesion mimic mutant, s*pl5*, Proteomics, 2-DE

## Abstract

**Background:**

A lesion-mimic mutant in rice (*Oryza sativa* L.), *spotted leaf 5* (*spl5*), displays a disease-resistance-enhanced phenotype, indicating that SPL5 negatively regulates cell death and resistance responses. To understand the molecular mechanisms of *SPL5* mutation-induced cell death and resistance responses, a proteomics-based approach was used to identify differentially accumulated proteins between the *spl5* mutant and wild type (WT).

**Results:**

Proteomic data from two-dimensional gel electrophoresis showed that 14 candidate proteins were significantly up- or down-regulated in the *spl5* mutant compared with WT. These proteins are involved in diverse biological processes including pre-mRNA splicing, amino acid metabolism, photosynthesis, glycolysis, reactive oxygen species (ROS) metabolism, and defense responses. Two candidate proteins with a significant up-regulation in *spl5* – APX7, a key ROS metabolism enzyme and Chia2a, a pathogenesis-related protein – were further analyzed by qPCR and enzyme activity assays. Consistent with the proteomic results, both transcript levels and enzyme activities of APX7 and Chia2a were significantly induced during the course of lesion formation in *spl5* leaves.

**Conclusions:**

Many functional proteins involving various metabolisms were likely to be responsible for the lesion formation of *spl5* mutant. Generally, in *spl5*, the up-regulated proteins involve in defense response or PCD, and the down-regulated ones involve in amino acid metabolism and photosynthesis. These results may help to gain new insight into the molecular mechanism underlying *spl5*-induced cell death and disease resistance in plants.

**Electronic supplementary material:**

The online version of this article (doi:10.1186/1939-8433-6-1) contains supplementary material, which is available to authorized users.

## Background

In plants, one of the most common and effective defense responses to pathogen attack is the hypersensitive response (HR), which prevents further spread of pathogens to adjacent cells (Morel and Dangl 1997). Lesion mimic mutants (*lmms*), displaying HR-like lesions in the absence of pathogen attacks, have been identified from maize (Johal et al. 1995), Arabidopsis (Dietrich et al. 1994), barley (Wolter et al. 1993), and rice (Takahashi et al. 1999). Most *lmms* constitutively activate immune responses, including callose deposition, induction of *Pathogenesis* *related* (*PR*) genes, production of reactive oxygen species (ROS), and accumulation of phytoalexins (Staskawicz et al. 1995). Therefore, *lmms* are very useful genetic tools to dissect molecular mechanisms of programmed cell death (PCD) and defense responses in plants.

In rice, more than 43 *lmms* have been isolated, most of which display enhanced resistance to rice blast and/or bacterial blight pathogens (Takahashi et al. 1999; Yin et al. 2000; Mizobuchi et al. 2002; Jung et al. 2005; Mori et al. 2007; Wu et al. 2008; Qiao et al. 2010). So far, at least 11 *lmms* have been functionally characterized, including *spl7* (Yamanouchi et al. 2002), *spl11* (Zeng et al. 2004), *Spl18* (Mori et al. 2007), *spl28* (Qiao et al. 2010), *sl* (Fujiwara et al. 2010), *ttm1* (Takahashi et al. 2007), *rlin1* (Sun et al. 2011), *NPR1* (Chern et al. 2005), *lsd1* (Wang et al. 2005), *acdr1* (Kim et al. 2009), and *edr1* (Shen et al. 2011). Interestingly, these *LMM* genes encode different proteins with distinct functions. For example, SPL7 is a heat stress transcription factor (Yamanouchi et al. 2002); SPL11 is a E3 ubiquitin ligase (Zeng et al. 2004); SPL18, a acyltransferase (Mori et al. 2007); SPL28, a clathrin-associated adaptor protein complex 1 medium subunit 1 (Qiao et al. 2010). These findings indicate that numerous proteins with distinct functions in multiple signaling pathways and/or processes are involved to prevent inappropriate activation of PCD. Thus, *lmms* have helped to gain an in-depth insight into regulatory mechanisms of PCD and defense responses in plants.

Rice *spotted leaf 5* (*spl5*) is a *lmm* with spontaneous HR-like lesions on its leaves, and broadly enhanced resistance to rice blast and bacterial blight pathogens (Yin et al. 2000; Mizobuchi et al. 2002). The *spl5* gene was previously mapped into a 36.4-cM region on rice chromosome 7 (Iwata et al. 1978). Recently, we finely mapped and isolated *spl5* by a map-based cloning, and surprisingly, it was found that the protein encoding by *SPL5* gene (GeneBank accessioin: KC128660) shares a certain degree of homology with a human splicing factor 3b subunit 3 (SF3b3), one subunit of the SF3 protein complex involved in binding of U2 snRNP to the branch site in the splicing reaction of pre-mature RNAs (Chen et al. 2009, Chen et al. 2012). Therefore, it is likely that the SPL5 regulated cell death and resistance responses post-transcriptionally.

Two-dimensional gel electrophoresis (2-DE) is a most commonly used proteomics technology for monitoring global changes in protein levels in plants (Agrawal and Rakwal 2006). The comparative proteomics has been used to identify differentially expressed proteins between wild type (WT) rice and *lmms* (Takahashi et al. 2003; Tsunezuka et al. 2005; Jung et al. 2006; Kang et al. 2007; Kim et al. 2008). However, different defense-related proteins and metabolic enzymes were found to be differently accumulated during lesion formation in a *lmm*-specific manner or in different *lmms*. For example, two PR proteins (OsPR5 and OsPR10) and three ROS-scavenging enzyames [catalase (CAT), ascorbate peroxidae (APX), and superoxide dismutase (SOD)] were differentially expressed in the *blm* mutant (Jung et al. 2006); Peroxidase, thaumatin-like protein, probenazole-induced protein (PBZ1) were up-regulated in the *spl1* mutant (Kim et al. 2008).

Here, we compared the protein profiles of *spl5* mutant and WT by 2-DE and found that 14 proteins were differentially accumulated between WT and *spl5*. Among these 14 proteins, 7 were up-regulated and 7 were down-regulated, respectively, in *spl5*. The proteins up-regulated in *spl5* are those involved in defense response or PCD, and the proteins down-regulated in *spl5* involved in amino acid metabolism and photosynthesis. Interestingly, a clear correlation between levels of protein accumulation and levels of gene expression (or induction) was observed for the 7 up-regulated proteins in *spl5*. However, a corresponding correlation was not observed for the 7 down-regulated proteins in *spl5*. Together, our results may help to understand molecular mechanisms of lesion formation in *spl5*.

## Results and discussion

### 2-DE analysis between WT and *spl5* mutant

**Figure 1:**
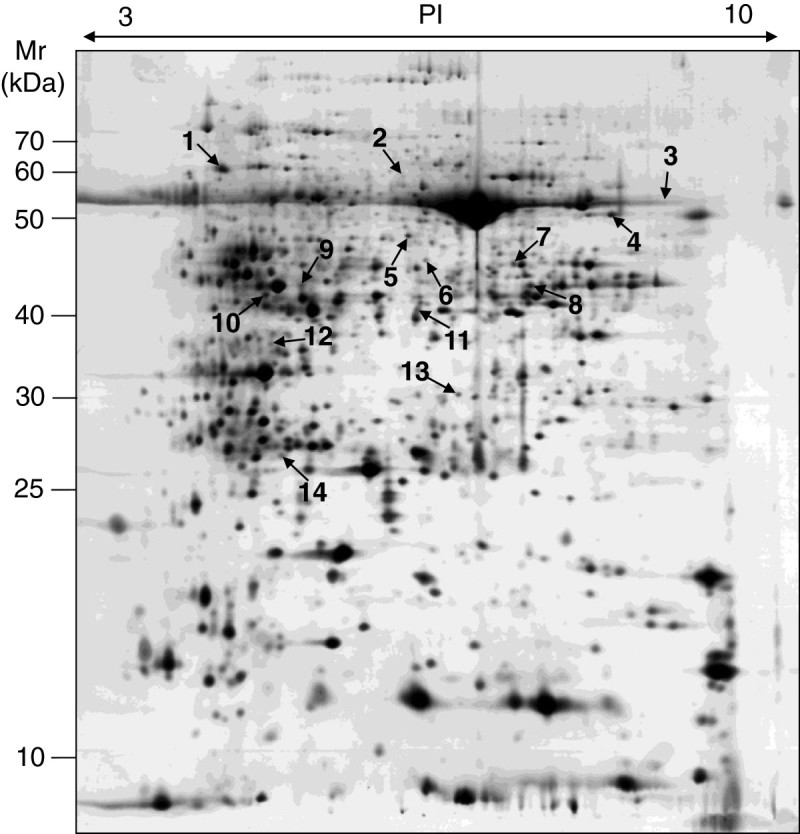
**A silver-stained 2-DE gel of the proteins extracted from leaf blades of both WT and** ***spl5*** **mutant.** For IEF, 100 μg of total proteins was loaded onto pH 3-10 IPG strips (13 cm, nonlinear), and then transferred to 12.5% SDS-polyacrylamide gel for the second-dimensional electrophoresis. The protein gel was stained with silver nitrate solution. Quantitative analysis of digitized images was carried out using the Image Master software (Amersham, USA). Arrows indicate spots with more than 2-fold change in *spl5* mutant compared to WT.

**Figure 2:**
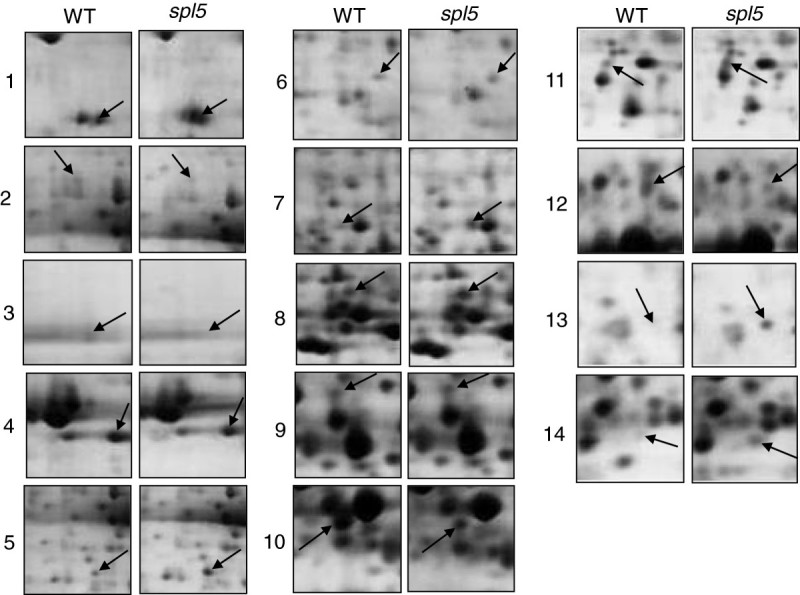
**Magnified regions of 2-DE gels.** Numbers at the left of images indicate protein spots showed by arrows in Figure 1 with significantly differential accumulation levels between *spl5* and WT mutant in 2-DE gel analysis.

To compare protein expression profiles between WT and the *spl5* mutant, total proteins extracted from fully developed leaves with lesions from *spl5* and the corresponding leaves from WT were analyzed by 2-DE. After quantitative analysis, 14 spots with > 2-fold changes (p < 0.05) between *spl5* and WT were identified (Figures 1 and 2). Compared to those in WT, seven proteins (spots 1, 5, 6, 8, 11, 13, and 14) were up-regulated and seven (spots 2, 3, 4, 7, 9, 10, and 12) were down-regulated, respectively, in *spl5* mutant. Relative intensities and fold-changes of the spots differentially expressed between *spl5* and WT were shown in Table 1.

**Table 1:** **Identification of proteins differentially expressed between WT and** ***spl5*** **mutant**

Function type	Spot id	Homologous protein	Score	Source	Accession	Coverage (%)	pI	MM (kDa)	Change fold	mRNA level
mRNA splicing	2	Thioredoxin-like protein 4B	71	*O. sativa*	gi|125576924	25	6.4	23.6	−3.3	-
Amino-acid metabolism	4	Alanine aminotransferase	187	*O. sativa*	gi|115470235	31	8	54.0	−2.3	-
	6	Aspartate aminotransferase	84	*O. sativa*	gi|125541475	15	6.5	50.6	2.0	up
	12	Cysteine synthase	86	*O. sativa*	gi|115489664	29	5.3	33.9	−3.9	-
	5	S-adenosylmethionine synthetase	93	*O. sativa*	gi|100801534	27	6.5	43.0	2.6	up
Photosynthesis	3	Rubisco large subunit	130	*O. sativa*	gi|115468792	33	8.5	48.4	−2.7	down
	9	Rubisco activase	74	*O. sativa*	gi|1778414	28	5.4	48.1	−2.0	-
	10	Rubisco activase, chloroplast precursor	174	*O. sativa*	gi|108864712	44	5.1	36.7	−3.1	-
Glycolysis	8	Glyceraldehyde-3-phosphate dehydrogenase	78	*O. sativa*	gi|115459078	24	7.3	36.9	3.1	up
ROS metabolism	11	Glutathione S-transferase 14	88	*O. sativa*	gi|46276327	33	6.5	30.8	2.1	up
	13	Ascorbate peroxidase 7	264	*O. sativa*	gi|116310282	47	6.9	38.2	8.1	up
Defense-related	14	Chitinase Chia2a	81	*O. sativa*	gi|115483206	27	5.4	27.9	3.5	up
Others	1	Retrotransposon Ty3-gypsy subclass	68	*O. sativa*	gi|77556153	16	4.7	29.1	3.0	down
	7	Nad-dependent formate dehydrogenase	164	*O. sativa*	gi|4760553	40	7.2	41.5	−2.2	-

Spot ID refers to the spot identity as given in Figure 2; Accession, protein accession in GenBank (http://www.ncbi.nlm.nih.gov/); Coverage %, the percentage of sequence coverage; pI, experimental isoelectric points; MM, experimental molecular masses; Change fold, the expression change fold of protein level in *spl5* mutant compared to WT; mRNA level, the expression change of mRNA level in *spl5* mutant compared to WT.

### Characterization of differentially expressed proteins in *spl5* mutant

The 14 differentially expressed spots represent 14 annotated proteins, which could be categorized into seven functional classes including pre-mRNA splicing, amino acid metabolism, photosynthesis, glycolysis, ROS metabolism, defense-related, and other processes (Table 1). Interestingly, these proteins are mostly associated with cell death and defense responses in different organisms.

#### Pre-mRNA splicing protein

A protein component of RNA spliceosome, thioredoxin-like protein 4B (TXNL4B, Dim2, or DLP), was down-regulated in the *spl5* mutant. In eukaryotes, the pre-mRNA splicing that removes intronic sequences is undertaken by the spliceosome, a macromolecular complex containing four snRNPs (U1, U2, U4/U6, and U5) and numerous auxiliary proteins (Kramer 1996). DLP functions in the cell nucleus and interacts with an U5 protein subunit of the spliceosome, and blocking DLP protein activity leads to insufficient pre-mRNA splicing (Sun et al. 2004).

#### Amino-acid metabolism enzymes

In differently expressed proteins, four enzymes including alanine aminotransferase (ALT; down), aspartate aminotransferase (AST; up), cysteine synthase (CSase; down), and S-adenosylmethionine synthetase (SAMS; up) (Table 1) are involved in amino acid transport and metabolism. SAMS catalyzes the biosynthesis of S-adenosylmethionine, which is a co-substrate for methylation reactions and serves as substrate for the synthesis of ethylene and the polyamines (Burstenbinder et al. 2010). Ethylene is an important hormone involved in plant responses to various stress situations (e.g. pathogen attack); and exposure of plants to ethylene can induce disease resistance (Geraats 2003). Polyamines can contribute to hydrogen peroxide (H_2_O_2_) formation in response to pathogen infections, which led to increased necrosis and resistance to disease (Marina et al. 2008; Moschou et al. 2009; Gonzalez et al. 2011). The up-regulation of SAMS was also found in the mutant *cdr2* (Tsunezuka et al. 2005).

#### Photosynthesis proteins

Rubisco is a critical enzyme involved in photosynthetic CO_2_ assimilation and photorespiratory carbon oxidation. Rubisco is inactivated by ROS, and degraded during senescence and oxidative stresses (Ranjan et al. 2001; Sedigheh et al. 2011). The reduction of the Rubisco large subunit (Rubisco-L) in *spl5* mutant might be caused by H_2_O_2_ over-accumulation (Chen et al. 2012). Moreover, in the present study we found that Rubisco activases (Rubisco-A), which catalyze Rubisco activation, were also down-regulated in *spl5* mutant (Table 1). The reduction of Rubisco and/or Rubisco-A was also found in the *lmms* of *spl1* (Kim et al. 2008), *spl6* (Kang et al. 2007), *crd2* (Tsunezuka et al. 2005), and *blm* (Jung et al. 2006), suggesting that the reduction in Rubisco and Rubisco-A accumulation is a shared phenomenon among *lmms*.

#### Glycolysis protein

A key enzyme of glycolysis, glyceraldehyde-3-phosphate dehydrogenase (GAPDH), was up-regulated in *spl5* mutant (Table 1), as also observed in *lmms spl1* (Kim et al. 2008) and *cdr2* (Tsunezuka et al. 2005). GAPDH catalyzes the oxidation of dihydroxyacetone phosphate to glycerol-3-phosphate. More recently, GAPDH emerged as a multifunctional protein in several non-metabolic processes, namely a primary role in apoptosis. S-nitrosylated GAPDH initiates apoptotic cell death by nuclear translocation following Siah1 binding (Hara et al. 2005); GAPDH accumulates in mitochondria during apoptosis and induces the pro-apoptotic mitochondrial membrane permeabilization (Tarze et al. 2007); and it also mediates cell death by its nuclear translocation under oxidative stress (Nakajima et al. 2009). Additionally, it was reported that *GLY1*-encoded GAPDH plays an important role in plastidal oleic acid-mediated signaling and resistant signaling in Arabidopsis (Kachroo et al. 2004,2005; Chandra-Shekara et al. 2007; Xia et al. 2009). Compared to WT, Arabidopsis mutant *gly1* is much more susceptible to pathogens, while plants with *GLY* *1* overexpression have enhanced resistance (Venugopal et al. 2009). Increased level of GAPDH protein in the *spl1*, *spl5*, and *cdr2* mutants suggests that GAPDH may function in stimulating cell death and defense responses in rice.

#### ROS metabolism proteins

Two major enzymes of ROS-detoxification, glutathione S-transferase 14 (GST14) and APX7, were up-regulated in *spl5* mutant (Table 1). ROS, such as superoxide anion (O_2_^–^) and H_2_O_2_, are toxic byproducts of aerobic metabolism (Mittler et al. 2004). Upon pathogen attack, ROS were immediately induced to kill the infected cells and also served as a signal to activate the defense response (Shigeoka et al. 2002). To avoid the oxidative damage to other cells in plants, the ROS must be scavenged by the antioxidant enzymes SOD, CAT, APX, or GST etc. H_2_O_2_ has been reported to up-regulate expression of *APX* (Lee et al. 1999; Morita et al. 1999). According to our previous results, H_2_O_2_ is over-accumulated in leaves of *spl5* mutant (Chen et al. 2012). It is likely that the high level of H_2_O_2_ in this mutant induces the HR and increases the resistance to pathogens. Therefore, up-regulation of APX7 and GST14 might be responsible for scavenging excessive accumulation of ROS in *spl5* mutant. However, inductions of APX7 and GST14 were apparently insufficient to detoxify the overproduction of ROS, which resulted in cell death in *spl5* mutant.

#### Defense-related protein

A PR protein, Chia2a, was found to be differently expressed between WT and *spl5* mutant (Table 1). Chia2a is a class II chitinase belonging to the PR-3 group (Muthukrishnan et al. 2001). Chitinase can break down glycosidic bonds in chitin, which is the main structural component of fungal cell walls and insect exoskeletons (Sela-Buurlage et al. 1993). The expression of the chitinase gene was significantly stimulated by fungi (Xu et al. 2008). Transgenic plants over-expressing chitinase gene showed enhanced resistance to fungal (Brogue et al. 1991; Dunsmuir et al. 1993; Oldach et al. 2001) and bacterial pathogens (Oldach et al. 2001). It is likely that the increased level of Chia2a is responsible, at least partially, for the enhanced resistance in *spl5* mutant.

### mRNA level of differentially expressed proteins in *spl5* mutant

**Figure 3:**
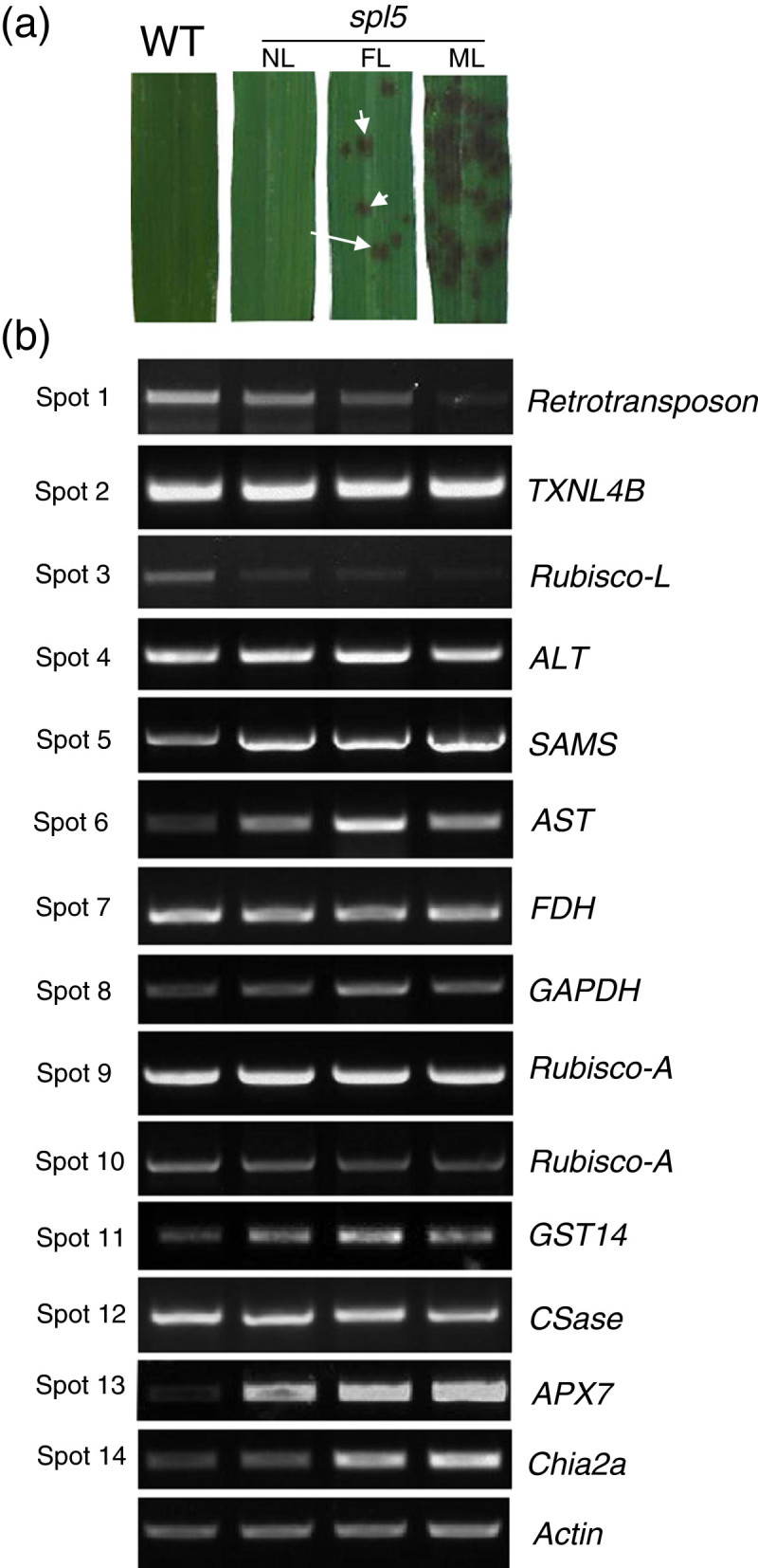
**Gene expressions of candidate proteins by semi-quantitative RT-PCR.** (**a**) Lesion-mimic phenotypes of *spl5* mutants. WT (control), the leaf blades of WT; NL, FL and ML indicate no lesion, few lesions, and many lesions, respectively, in the leaf blades of *spl5* mutant. Arrows indicate leaf lesions in *spl5* mutant. (**b**) Semi-quantitative RT-PCR of 14-proteins’ genes in WT leaves and leaf parts with different lesions of *spl5* mutant. The protein ID numbers are listed in the left of images, and the corresponding gene names are listed in the right of images. The *Actin* was used as a reference gene.

To assay the mRNA levels of 14 differentially expressed proteins in *spl5* mutant, the semi-quantitative RT-PCRs were performed. We analyzed the expressions of these 14 genes in WT leaf blades and the three different parts of *spl5* leaf blades, based on the degree of lesion formation: no lesion (NL), leaf area without any lesions; few lesions (FL), leaf area with 10–20% lesions; and many lesions (ML), leaf area with 70–80% lesions (Figure 3a). As shown in Figure 3b, in *spl5* mutant, the expression of 6 genes were induced and 2 genes were suppressed, and 6 genes did not changed at mRNA level.

We compared the genes expression profile to our 2-DE data (Table 1). Most of the 7 up-regulated proteins (except spot 1) were transcriptionally induced in *spl5* mutant. As expected, many of them (*SAMS*, *GAPDH*, *GST14*, *APX7* and *Chia2a*) are defense- or PCD-related. It is likely that the SPL5 protein negatively regulates expression of these genes at transcriptional level. In contrast, the genes encoding down-regulated proteins (except spot 3) did not change significantly at the transcriptional level in *spl5* mutant, and most of these proteins involve in amino-acid metabolism and photosynthesis. Based on the fact that the *SPL5* gene encodes a subunit of splicing factor (Chen et al. 2012), it is likely that the genes encoding these down-regulated proteins might be controlled directly or indirectly by SPL5 through the post-transcriptional mRNA processing.

### Further analysis of APX7 and Chia2a in *spl5* mutant

**Figure 4:**
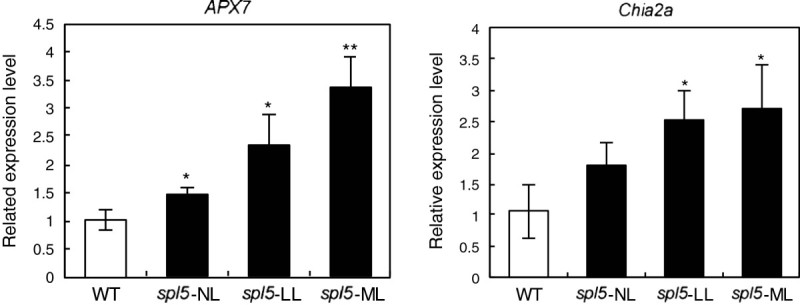
**qPCR confirmation of gene expression for** ***APX7*** **and** ***Chia2a*** **.** qPCR results for *APX7* and *Chia2a* in the leaf blades of WT (control) and leaf parts with different degree of lesions of *spl5* mutant (see Figure 3a). Single and double asterisks indicate P < 0.05 and P < 0.01 (Student’s *t*-test), respectively.

**Figure 5:**
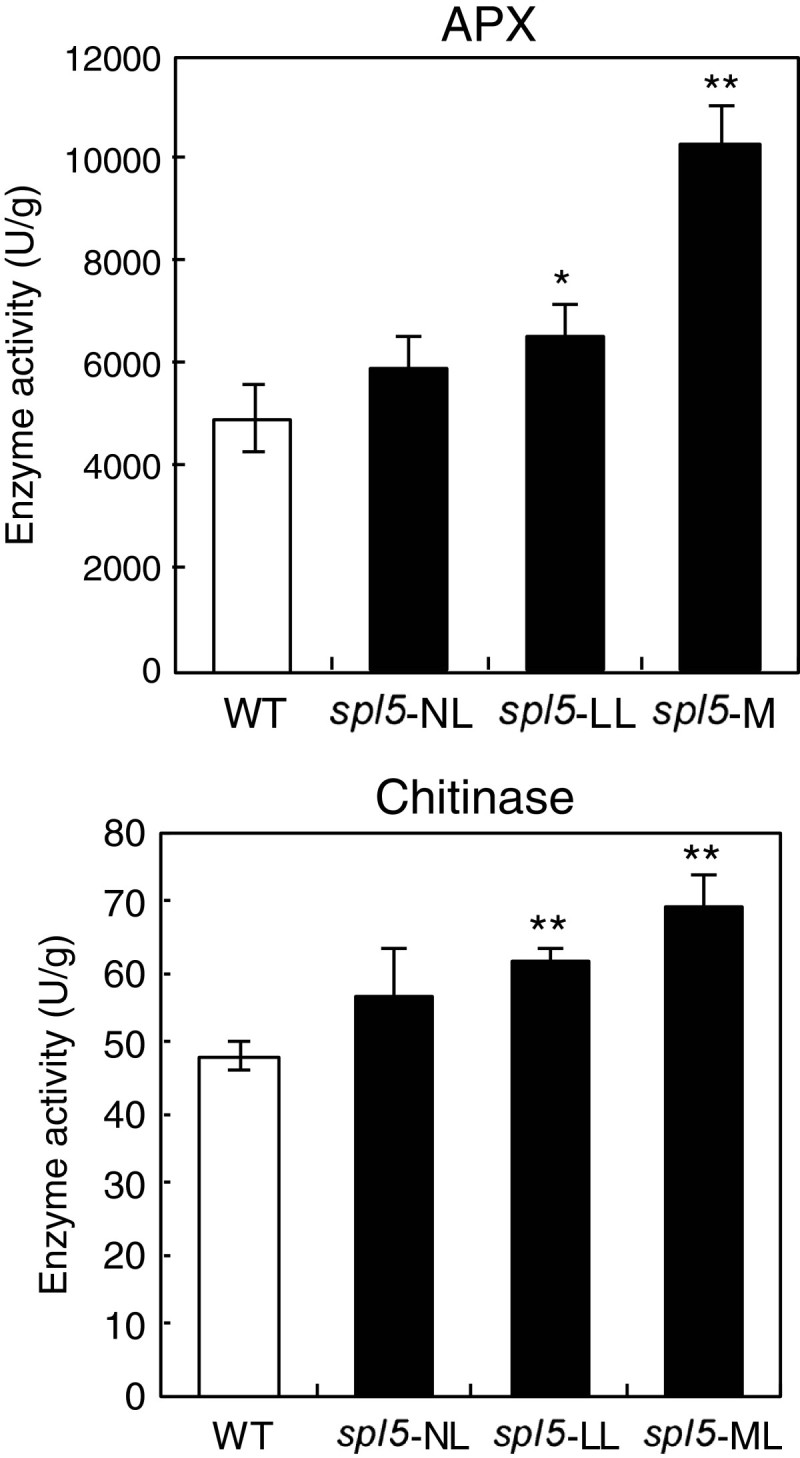
**Enzyme activities of APX and Chitinase in** ***spl5*** **mutant.** Leaf parts with different number of lesions of *spl5* mutant (see Figure 3a) were used to analyze enzyme activity: (**a**) APX and (**b**) chitinase. Single and double asterisks indicate P < 0.05 and P < 0.01 (Student’s *t*-test), respectively.

Since the APX7 and Chia2a are directly involved in mediating PCD or defense responses, the gene expression of these proteins were further confirmed by qPCR. The qPCR results were similar to that of semi-quantitative RT-PCR (Figure 3b and Figure 4). The expression of *APX7* increased in NL, FL, and ML of *spl5* leaves, while the expression of *Chia2a* increased only in FL and ML but not in NL (Figure 4). The expression levels of these two genes were positively correlated with lesion numbers on the leaves of the *spl5* mutant. Moreover, we further examined enzyme activities of APX and chitinase both in WT and *spl5* mutant. As expected, APX and chitinase activities were proportional to the number of lesions on *spl5* leaves (Figure 5).

## Conclusions

According to our 2-DE data and the proteomic results of other rice *lmms* (Takahashi et al. 2003; Tsunezuka et al. 2005; Jung et al. 2006; Kang et al. 2007; Kim et al. 2008), proteins involved in ROS scavenging, defense responses or cell death such as SOD (*spl1*, *blm*), CAT (*spl6*, *blm*), Peroxidase (*spl1*), APX (*spl5*, *blm*), GST (*cdr2*, *spl5*), were likely to be induced in *lmms*, whereas proteins involved in photosynthesis and glycolysis such as Rubisco (*spl1*, *spl5*, *spl6*, *blm*, *crd2*), Rubisco-A (*spl5*, *spl6*), and GAPDH (*spl1*, *spl5*, *cdr2*) were often deceased in *lmms*; defense-related protein like OsPR5 (*spl1*), OsPR10 (*blm*), PBZ1 (*spl1*, *cdr2*, *blm*), Chia2a (*spl5*) were significantly activated in *lmms*. This suggests that the differential accumulation of these proteins is common features for *lmms*.

## Methods

### Plant materials and growth conditions

An original *spl5* mutant was screened from a γ-radiation-mutagenesis population of Norin8 (*Oryza sativa* L. ssp. *japonica*) by Iwata et al. (1978) in Japan. Zeng et al. (2003) crossed the *spl5* mutant with WT Zhefu802 (a Chinese *indica* cultivar) through repeated backcrossing, and produced the mutant Zhefu802^*spl5*/*spl5*^ with *spl5* lesion mimic phenotype as used in this study. The seeds of *spl5* mutant and Zhefu802 were germinated in an incubator at 28°C and then incubated in nutrient solution (Yoshida et al. 1976) in a growth chamber at 28/24°C (day/night). The nutrient solution was maintained at pH 5.6 and refreshed each 5 d. The fully developed leaves of 60-days-old seedlings were collected from each plant and immediately frozen in liquid nitrogen and stored at −80°C.

### Protein extraction

Total proteins were extracted from collected leaf samples of 10 plants and independently repeated three times. Leaf blades were ground in liquid nitrogen, and the tissue powder produced was immediately suspended in an extraction buffer containing 9.5 M urea, 4% w/v 3-[(3-cholamidopropyl) dimethylammonio]-1-propane-sulfonate (CHAPS), 65 mM dithiothreitol (DTT), and 2% v/v immobilized pH gradient (IPG) buffer pH 3–10. Crude homogenates were centrifuged at 4°C (9,000 × *g* for 30 min). The supernatants were precipitated by 10% TCA for 1 h at −20°C, followed by centrifugation at 13,000 × *g* for 30 min. The pellets were washed twice with cold acetone and allowed to air dry, and then resuspended with the extraction buffer and finally stored at −80°C. Protein contents were determined by the Bradford method (Bradford 1976) using a protein assay reagent (Bio-Rad, USA).

### 2-DE

Extracted proteins were analyzed by 2-DE and biologically repeated trice using different samples. For 2-DE, 100 and 500 μg of total proteins were loaded onto analytical and preparative gels, respectively. The Ettan IPGphor Isoelectric Focusing System (Amersham, USA) and pH 3–10 IPG strips (13 cm, nonlinear; Amersham) were used for isoelectric focusing (IEF). The IPG strips were rehydrated for 12 h in 250 μL of rehydration buffer containing the protein samples. IEF was performed in five steps: 30 V for 12 h, 500 V for 1 h, 1000 V for 1 h, 8000 V for 8 h, and 500 V for 4 h. The gel strips were equilibrated for 15 min in equilibration buffer [50 mM Tris–HCl (pH 8.8), 6 M urea, 2% sodium dodecyl sulfate (SDS), 30% glycerol, and 1% DTT]. This step was repeated using the same buffer with 4% iodoacetamide in place of 1% DTT. The strips were then subjected to the second-dimensional electrophoresis after transfer onto 12.5% SDS-polyacrylamide gels. Electrophoresis was performed using the Hofer SE 600 system (Amersham) at 15 mA per gel until the bromophenol blue reached the end of the gel. Both the proteins of WT and *spl5* mutant were done 2-DE for 3 gels, respectively.

### Gel staining and image analysis

After 2-DE, analytical gels were stained with ammoniacal silver nitrate based on the procedure described by Hochstrasser (1988), and preparative gels were stained with Coomassie Blue G250 (Bio-Rad). Resulting 2-D gels were scanned using an UMax Powerlook 2110XL Scanner (Amersham). The stained protein spots were detected using software Image Master (Amersham). After quantitative detection, the intensities of each spot were normalized by total valid spot intensity. The spots displaying significant changes were considered to be differentially expressed proteins. Expression differences per protein spot between the *spl5* mutant and WT from 3 independent experiments were estimated by *t*-test (p < 0.05). Protein spots were selected based on the significant differences of spots quantities between the *spl5* mutant and WT.

### In-gel digestion

Protein spots were excised from preparative 2-DE gels and destained with 100 mM NH_4_HCO_3_ and 30% acetonitrile (ACN). After removing the destaining buffer, the gel pieces were lyophilized and rehydrated in 30 μL of 50 mM NH_4_HCO_3_ containing 50 ng of sequencing grade, modified trypsin (Promega, USA). After digestion overnight at 37°C, these peptides were extracted thrice with 0.1% trifluoroacetic acid (TFA) in 60% ACN, and extracts were pooled together and lyophilized. Peptide mixtures were redissolved in 0.1% TFA, desalted and concentrated using ZipTips from Millipore. Peptide solution (0.75 mL) was mixed with 0.75 mL of matrix [α-cyano-4-hydroxycinnamic acid (CHCA) in 30% ACN, 0.1% TFA] spotted on a target disk and allowed to air dry.

### MALDI-TOF/TOF analysis and database searching

Mass spectra were acquired on a MALDI-TOF/TOF mass spectrometer, the Bruker-Daltonics AutoFlex TOF-TOF LIFT (Bruker, Germany). Protein database searching was performed with the MASCOT search engine (http://www.matrixscience.com) using monoisotopic peaks against the NCBI nonredundant protein database (http://www.ncbi.nlm.nih.gov/). The species selected was *Oryza sativa*.

### Semi-quantitative RT-PCR and qPCR

Total RNAs of leaves were isolated by TRIzol Reagent (Invitrogen, USA). The first-strand synthesis of cDNAs was carried out by SuperScript® III First-Strand Synthesis System (Invitrogen) according to the manufacturer’s instruction. Semi-quantitative RT-PCR was performed for 25 cycles of 30 s at 94°C, 30 s at 60°C, and 1 min at 72°C. qPCR was performed in StepOne™ Real-Time PCR System (Applied Biosystems, USA) using the Fast SYBR Green Master Mix reagent (Applied Biosystems) by the manufacturer’s instructions, and the thermal cycle used was as follows: 95°C for 20 s; and 40 cycles of 95°C for 3 s, and 60°C for 30 s. *OsRAc1* (GenBank accession: X16280), a rice constitutively expressed gene *of Actin*, was used as a standardization control, using the primer pair 5’-GGAACTGGTATGGTCAAGGC-3’ and 5’-AGTCTCATGGATACCCGCAG-3’ for semi--quantitative RT-PCR, 5’-TGGCATCTCTCAGCACATTCC-3’ and 5’-TGCACAATGGATGGGTCAGA-3’ for qPCR. Gene-specific primers of candidate genes for semi-quantitative PCR and qPCR are listed in Table 2 and Table 3, respectively. Independent biological repetitions of each experiment were performed three times.

**Table 2:** **Gene-specific primers for RT-PCR in this study**

^a^Spot id	^b^Accession	Forward primer (5’-3’)	Reverse primer (5’-3’)	^c^ Tm (°C)
1	LOC_Os12g37540	AACAAGGTAGGGATAGTTACTT	CCTTGTATGTGGGTTTTTTAGAA	55
2	AK067692	AATGAAATCTTGCTTGCTGC	CTAAATCTTCTTGGGACATA	60
3	AK067692	GCCTACTTCTTCACATTCAC	ATTTCATTACCTTCACGAGC	55
4	AK067732	ACCCGCTTTATTCTGCTG	ATCCTTTTGACACAGTATGG	60
5	AK104875	AGATGCTGTGCTTGATGCCT	CAATGACGAAGCGACCAGAT	55
6	AK105059	CAAACAGGGTGAAGAGCCAG	CTCGCATTTAGCCAGGGACA	62
7	AK065872	ACGCCGACAAGAATCCCAAC	CAATACGACCAGCCCCAACA	60
8	AK064960	TTCATCACCACCGACTACAT	AACCCTCAACAATACCAAAC	55
9	AK060847	GAAGCTGAAGAAGCAGGTGACATC	CGAAGACGAGCTCACACTGGAAG	55
10	AK104332	ATGGGTGAATTCTGTGGTGAG	CCCTTCTTGATGATGTCTGCC	58
11	AK102889	CTCGTTGCGGTAGTGCTGCT	AATGAAATCTTGCTTGCTGC	55
12	AK099598	TGGCAGCGAAGACAAACAAC	CTGGAAGAGCACCGACGAAA	62
13	AK063934	GCTTGAGATTTGATGTTGAG	GTCCTCTGCGTATTTTTCTG	62
14	AK070067	CGACTTCTCCACCCTACTAT	ATGATGTTGGTGATGACGCC	55

^a^ Spot identity of protein in 2-D gel image; ^b^mRMA accession of the corresponding protein in GenBank (http://www.ncbi.nlm.nih.gov/), except for spot 1 protein whose gene accession was only deposited in TIGR database (http://rice.plantbiology.msu.edu/index.shtml); ^c^Anneal temperature of primer pair used in RT-PCR.

**Table 3:** **Gene-specific primers for qPCR in this study**

Gene name	Forward primer (5’-3’)	Reverse primer (5’-3’)
*APX7*	ATACGCAGAGGACCAAGAAGCAT	CTACGAGCAAGATAAATAGCAGA
*Chia2a*	CCAACATCATCAACGGCGGCAT	TTGGGATACTACATCACTACAT

### Assay of enzyme activities

About 0.5–1 g of leaves were homogenized for activity assay of APX or chitinase, according to the methods previously described by Mishra et al. (1993) and Boller et al. (1983), respectively. Each experiment was independently repeated three times.

## Authors’ original submitted files for images

Below are the links to the authors’ original submitted files for images. Authors’ original file for figure 1 Authors’ original file for figure 2 Authors’ original file for figure 3 Authors’ original file for figure 4 Authors’ original file for figure 5 Authors’ original file for figure 6 Authors’ original file for figure 7 Authors’ original file for figure 8

## Abbreviations

2-DE: Two-dimensional gel electrophoresis
APX: Ascorbate peroxidase
CAT: Catalase
FL: Few lesions
GAPDH: Glyceraldehyde-3-phosphate dehydrogenase
GST: Glutathione S-transferase
HR: Hypersensitive response
IEF: Isoelectric focusing
Lmm: Lesion mimic mutant
ML: More lesions
NL: No lesion
PBZ1: Probenazole-induced protein
PCD: Programmed cell death
PR: Pathogenesis-related
ROS: Reactive oxygen species
SAMS: S-adenosylmethionine synthetase
SF3b3: Splicing factor 3b subunit 3
SOD: Superoxide dismutase
*spl*: *spotted leaf*
TXNL4B: Thioredoxin-like protein 4B
WT: Wild-type.
