# Supplementary figures and images for: Proteomic analysis of a disease-resistance-enhanced lesion mimic mutant spotted leaf 5 in rice
Source: Rice (N Y). 2013 Jan 7;6:1. doi: 10.1186/1939-8433-6-1 (PMC5394886; doi:10.1186/1939-8433-6-1)

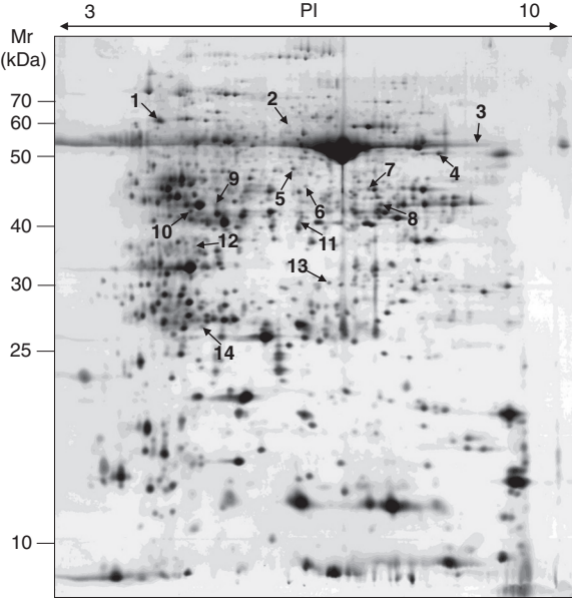

Supplement: Supplementary file 1 — Authors’ original file for figure 1 [file 12284_2012_39_MOESM1_ESM.pdf]

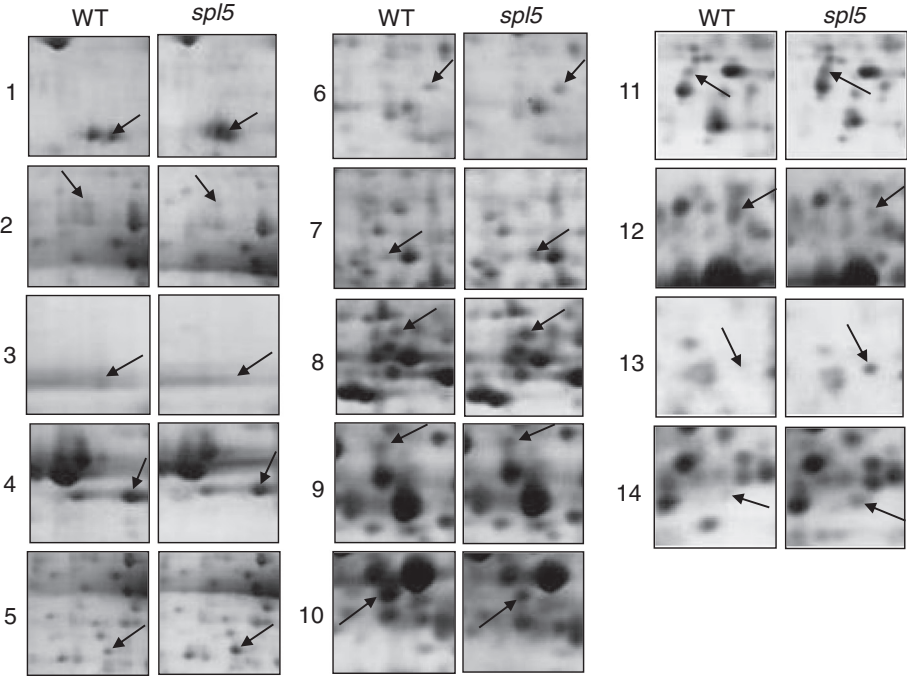

Supplement: Supplementary file 2 — Authors’ original file for figure 2 [file 12284_2012_39_MOESM2_ESM.pdf]

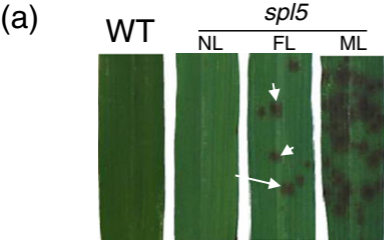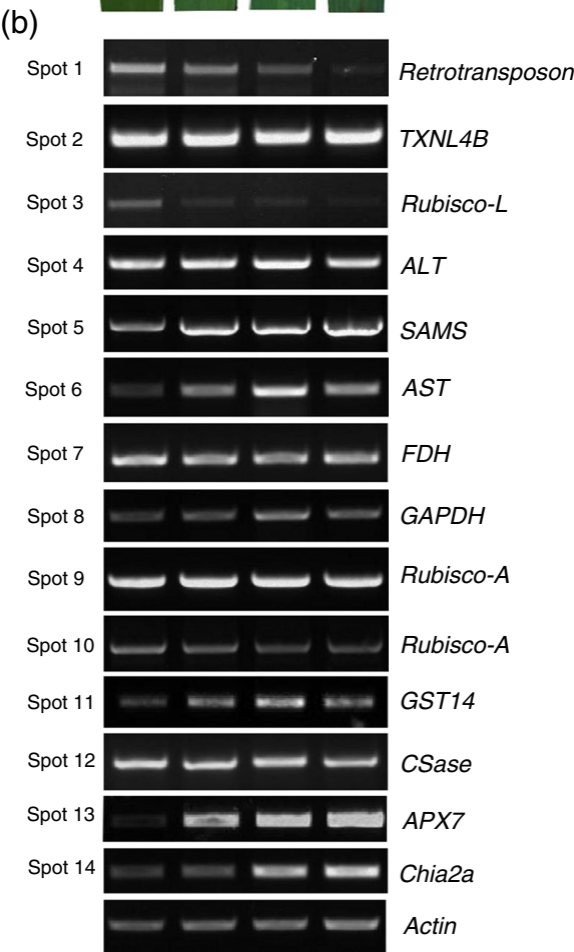

Supplement: Supplementary file 3 — Authors’ original file for figure 3 [file 12284_2012_39_MOESM3_ESM.pdf]

*APX7*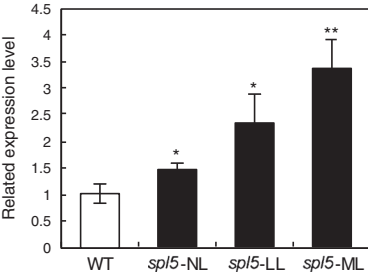*Chia2a*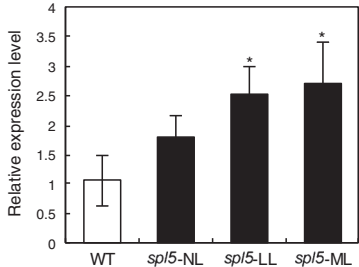

Supplement: Supplementary file 4 — Authors’ original file for figure 4 [file 12284_2012_39_MOESM4_ESM.pdf]

## APX

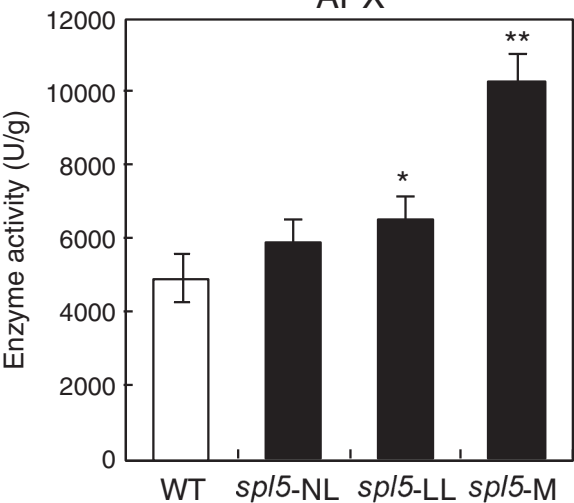

## Chitinase

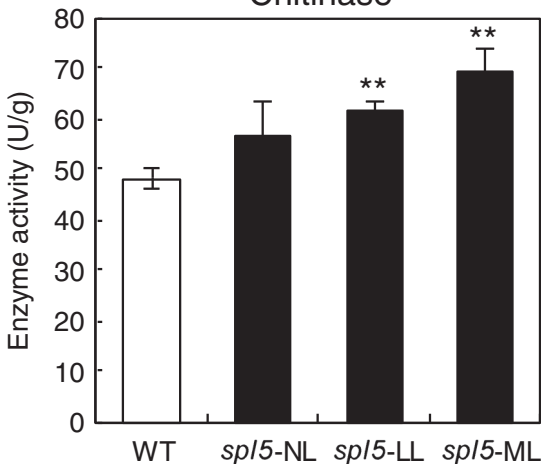

Supplement: Supplementary file 5 — Authors’ original file for figure 5 [file 12284_2012_39_MOESM5_ESM.pdf]

## Slide 1
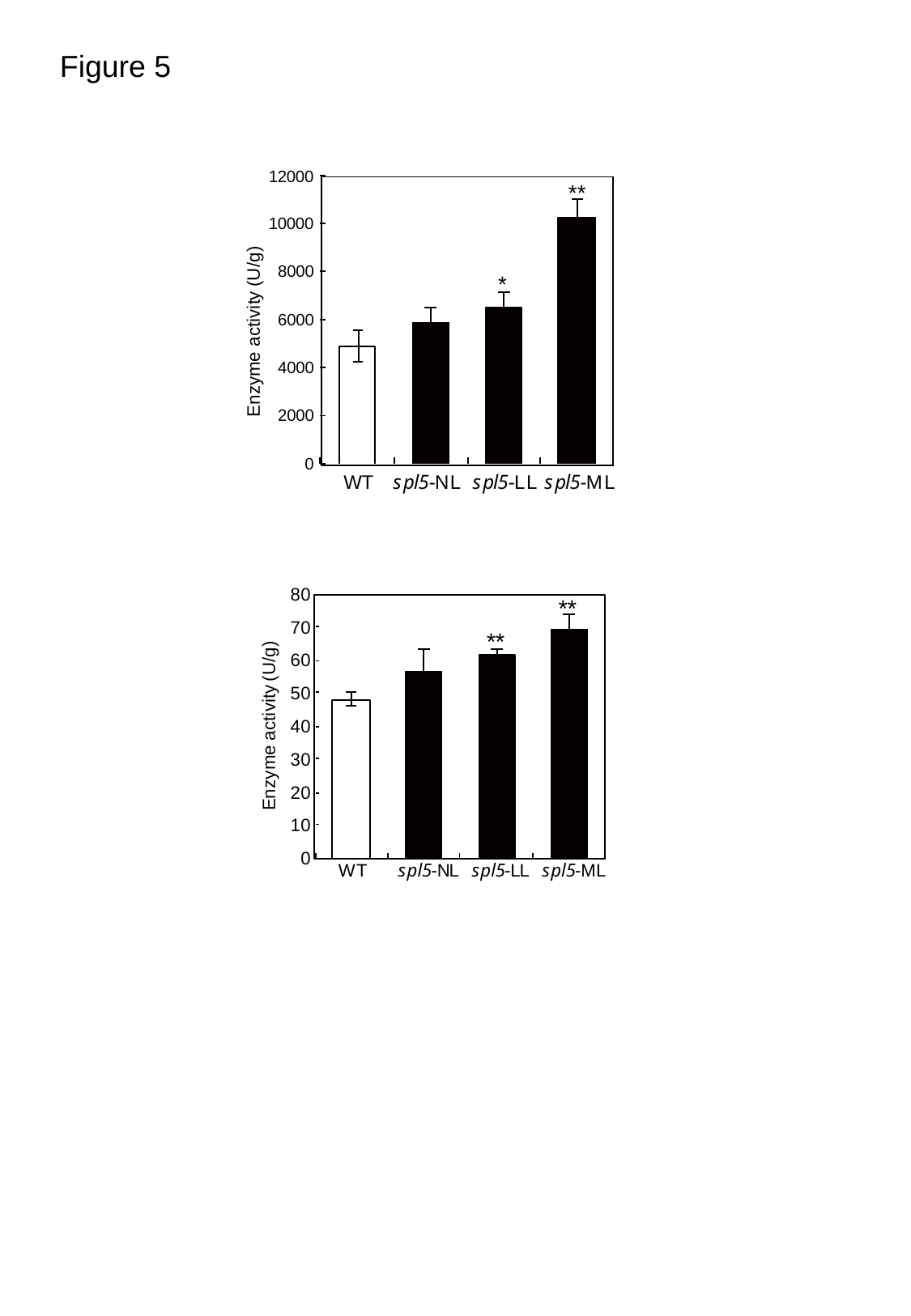

Figure 5

Supplement: Supplementary file 6 — Authors’ original file for figure 6 [file 12284_2012_39_MOESM6_ESM.ppt]
